# Crystal Nucleation in Ibuprofen Glass: Possible Relevance between the Characteristic Length of the Cooperatively Rearranging Region and the Size of Crystal Nuclei

**DOI:** 10.1021/acs.jpcb.4c07005

**Published:** 2025-02-06

**Authors:** Kohsaku Kawakami, Kaoru Ohyama

**Affiliations:** † Research Center for Macromolecules and Biomaterials, National Institute for Materials Science, 1-1 Namiki Tsukuba, Ibaraki 305-0044, Japan; ‡ Graduate School of Pure and Applied Sciences, University of Tsukuba, 1-1-1 Tennodai Tsukuba, Ibaraki 305-8577, Japan

## Abstract

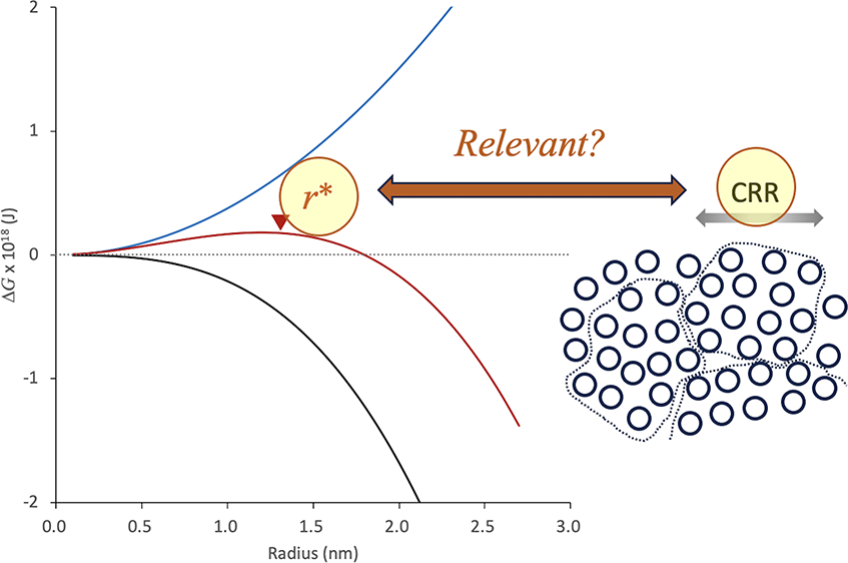

Crystallization behavior of ibuprofen glass was investigated with focus on the nucleation process and its possible relevance to the cooperatively rearranging region (CRR). The nucleation temperature range of ibuprofen glass was determined by annealing it at various temperatures, followed by observation of the probability of cold crystallization. The temperature to provide the highest probability of nucleation was −15 °C. The effect of the addition of a polymer was also investigated to find that it enhanced and suppressed the crystallization depending on the polymer species and its amount added. The added polymer seemed to influence both nucleation and crystal growth processes by decreasing the glass/nuclei interfacial tension and increasing viscosity, respectively. In addition, the coincidence of the size of CRR in the presence of the polymer with the critical size of nuclei was assumed to enhance nucleation. This finding provides a novel viewpoint for clarifying the nucleation mechanism from supercooled liquids and glasses.

## 1 Introduction

Drug molecules are typically in a crystalline state in solid pharmaceutical products. However, crystalline drugs are sometimes intentionally transformed into an amorphous state,^1−4^ which possesses higher Gibbs energy than the crystalline state. As its solubility is also higher,^5^ amorphization is an important strategy for the development of poorly soluble drugs. To prevent the crystallization of amorphous drugs during storage, it is of paramount importance to understand and control their crystallization behavior. Crystallization involves two steps: nucleation and crystal growth. Although many techniques, including X-ray diffraction and differential scanning calorimetry (DSC), are available for quantitatively evaluating the crystal growth process, direct detection of nucleation remains difficult.

The ease of crystallization depends on the compounds and is called the crystallization tendency or glass-forming ability.^6,7^ One of the most effective methods for evaluating the crystallization tendency of a compound is to determine the critical cooling rate required to inhibit crystallization during cooling from the liquid state.^6−8^ Another method for evaluating the crystallization tendency, particularly in the pharmaceutical field, involves subjecting the melt to a cooling/heating cycle to observe the crystallization behavior.^6,7,9^ If the compound crystallizes during cooling or during the subsequent reheating process, then it is classified as a Class I or II compound, respectively. Class III compounds do not crystallize during the cooling/reheating process. Most commercialized compounds in the amorphous state belong to this class,^10^ and a higher amount of polymer is included for ensuring sufficient stability margin.

The nucleation and crystal growth temperatures of higher-class compounds can significantly differ.^11^ In this case, nuclei may be formed without any indication of crystal growth during storage, which cannot be detected by conventional characterization techniques such as powder X-ray powder diffraction. The determination of the nucleation temperature is important, as amorphous solids with nuclei are much more physically unstable than those without nuclei.^12,13^ One method to determine the nucleation temperature of glass involves annealing at various temperatures, followed by heating using DSC, as the presence of nuclei promotes crystal growth.^14−16^ Thus, the nucleation temperature can be determined by observing the effect of annealing on the onset temperature and enthalpy of cold crystallization. However, this procedure is difficult to apply to compounds with low crystallization tendencies. In scientific papers, solid state crystallization is reported as if it occurs in a reproducible manner. However, for the compounds with low crystallization tendencies, crystallization behavior is not reproducible because of the stochastic nature of nucleation. In such cases, it should be more appropriate to analyze the result based on statistical thermodynamics, where the probability of phenomenon is related to entropy.

In this study, the crystallization of ibuprofen (IBP) glass is investigated. As this compound belongs to Class III in the crystallization tendency classification, its fresh glass does not crystallize during DSC heating. However, it can crystallize after annealing at the nucleation temperature. Thus, in the earlier study, it was subjected to the annealing study to find its nucleation temperature between −40 and −10 °C.^14^ However, as crystallization of IBP does not occur in a reproducible manner, focus was kept on the probability of crystallization during DSC heating of IBP glass in this study for identifying its nucleation temperature. This entropic approach does not ignore the low reproducibility problem of the crystallization behavior of IBP glass but rather is a logical way to deal with the issue.

Moreover, the effect of adding a small amount of polymer on the crystallization behavior was investigated based on the same approach. The polymer addition usually stabilizes the glass state; however, we found that destabilization can also occur. For explaining this unusual behavior, possible relevance between sizes of the crystal nuclei and the cooperatively rearranging region (CRR) is discussed.

## 2 Experimental Section

### 2.1 Materials

Racemic IBP was purchased from Fujifilm Wako (Osaka, Japan) and used without further purification. Vinylpyrrolidone-vinyl acetate copolymer (Kollidon VA64, PVPVA), Eudragit (poly(methacrylic acid-*co*-methyl methacrylate)) L100 (Eud), and hydroxypropyl methylcellulose acetate succinate (MG grade) (HPMCAS) were supplied from BASF (Ludwigshafen am Rhein, Germany), Evonik (Essen, Germany), and Shin-Etsu Chemical (Tokyo, Japan), respectively. All of the compounds were used as provided.

### 2.2 Preparation of IBP Glass and Investigation of the Nucleation Temperature

DSC (Q2000, TA Instruments, New Castle, DE, USA), calibrated by using indium and sapphire, was used to prepare and evaluate the IBP glass. Dry nitrogen was used as the inert gas at a flow rate of 50 mL/min. Tzero aluminum pans were used for the preparation and investigation, and approximately 3 mg of the crystalline IBP was loaded and sealed. Subsequently, the sample was heated to 90 °C at a rate of 10 °C/min for melting. After maintaining the temperature at 90 °C for 1 min, the sample was cooled at a rate of 20 °C/min to an annealing temperature. After annealing for 20 min or 1 h, the sample was reheated at 10 °C/min to observe cold crystallization unless otherwise mentioned. The measurements were repeated more than ten times for most conditions to determine the probability of crystallization.

### 2.3 Density Measurement

The true density of the IBP glass was determined on an AccuPyc II gas pycnometer (Micromeritics, Norcross, GA, USA) by using helium gas. As the glass transition temperature of IBP is well below room temperature, its amorphous state is not available in powder form. Thus, crystalline IBP was mixed with an equal amount of mesoporous silica material (Sylysia 320, Fuji Silysia Chemical, Kasugai, Japan) and melted at 100 °C, followed by cryomilling using a hand-shaking agate mill with liquid nitrogen. Density of the mixture was measured 10 times to obtain the mean value. The density of the mesoporous silica was determined to be 1.49 g/cm^3^. Using this value, the density of IBP was calculated.

### 2.4 Preparation of the IBP/Polymer Binary Mixture and Investigation of the Nucleation Temperature

IBP and the polymer (Eud, PVPVA, or HPMCAS) were mixed using a mortar and pestle at a designated mixing ratio and loaded into Tzero pans. The same temperature program used for pure IBP was used for the evaluation. More than ten samples were subjected to the same measurements.

### 2.5 Microscopic Observation of Crystal Growth

The growth process of the IBP crystal was investigated using polarized light microscopy (PLM) (Olympus BX-51, Tokyo, Japan) equipped with a U-POT polarizer and a U-ANT analyzer. IBP and its mixture with polymers were melted on thin glass using a hot plate heated at 100 °C, followed by cooling at ambient temperature and annealing at −20 °C for 1 h in a freezer. The samples were protected by moisture by storing them in an airtight box during the annealing in the freezer. Then, a cover glass was placed on the sample to investigate under PLM. The crystal growth behaviors were investigated at 40 °C. The sample temperature was controlled by a PN121-D heat stage (MSA Factory, Tokyo, Japan). Absence of crystals was confirmed before starting the investigation.

### 2.6 Determination of the Glass Transition Temperature and Characteristic Length of CRR

IBP and IBP/polymer mixtures were subjected to temperature-modulated DSC measurements to determine the glass transition temperature (*T*_g_) and characteristic length of CRR. The instrumental conditions are the same with those for the study to find the nucleation temperature, except that the sample amount was approximately 5 mg. After the melting at 90 °C, the sample was cooled at a rate of 20 °C/min to −60 °C. Then, the samples were heated in modulation mode at 2 °C/min with a 60 s period and 0.5 °C amplitude. The characteristic length of CRR, *L*, was determined by the following equation.^17,18^
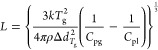
1where Δ*d*_*T*_g__ is half of the glass transition width; ρ is the density, which was determined as 1.09 g/cm^3^ for the fresh IBP glass; and *k* is the Boltzmann constant. Although this value was determined using a mesoporous silica carrier, it agreed well with the simulated value.^19^*C*_pg_ and *C*_pl_ are the heat capacities of the glass and super-cooled liquid, respectively.

### 2.7 Fourier-Transform Infrared (FT-IR) Spectroscopy

FT-IR spectra were acquired on a Jasco 6200 spectrophotometer (JASCO Corp, Tokyo, Japan) equipped with an ATR stage under a flow of dry nitrogen. The physical mixtures of IBP and the polymer were subjected to melting in DSC as described above. After quenching, the samples were collected from the Tzero pans to be subjected to the measurements. All spectra were recorded at 4 cm^–1^ intervals.

### 2.8 Determination of Fragility

Fragility, *m*, was determined from the dependence of *T*_g_ on the ramp rate, *q*, using DSC. The following equation was used for the calculation.^20^
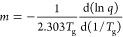
2

The cooling rate is generally set equal to the heating rate to observe *T*_g_ for calculating fragility; however, this procedure posits strong constraints, as slow cooling rates induce crystallization in most low-molecular-weight organic compounds. Therefore, a constant cooling rate of 20 °C/min and the middle point *T*_g_ were used as verified previously.^20^

## 3 Results and Discussion

### 3.1 Crystallization Tendency of IBP

**Figure 1:**
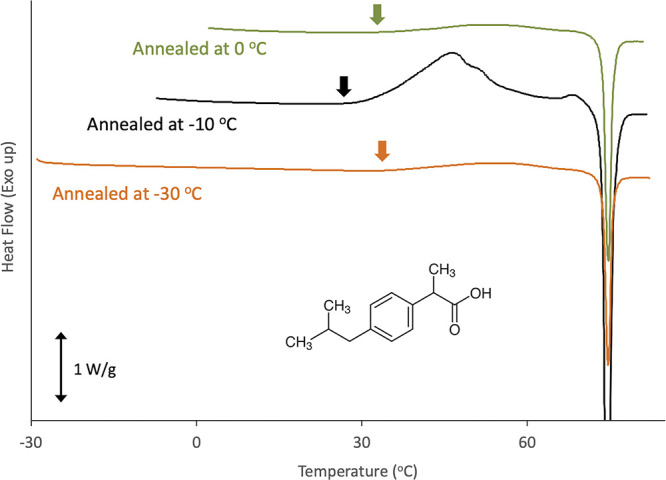
Examples of the DSC heating curves of IBP glasses after annealing at −30, −10, and 0 °C for 1 h. The arrows indicate the onset points of the cold crystallization peaks. The chemical structure of IBP is indicated in the figure.

IBP is a Class III compound in the classification system of the crystallization tendency, which means that cold crystallization cannot be observed during the heating of its glass using DSC.^7^ Thus, Dudognon et al. performed isothermal annealing for 6 h at various temperatures to induce nucleation, followed by observation of cold crystallization in the DSC heating curves. They concluded that the nucleation temperature of IBP glass for Form I was in the temperature range −40 to −10 °C.^14^ However, cold crystallization was not consistently observed after the 1 h annealing. Examples of the DSC heating curves of the IBP glass after annealing at various temperatures for 1 h are presented in the Supporting Information. Figure 1 shows examples of the curves where cold crystallization was observed. Both the onset crystallization temperature and crystallization enthalpy were influenced by the annealing temperature. Cold crystallization occurred in the temperature range of 50 to 80 °C, which could be assumed as the temperature range of crystal growth.

### 3.2 Nucleation Temperature of IBP Glass

**Figure 2:**
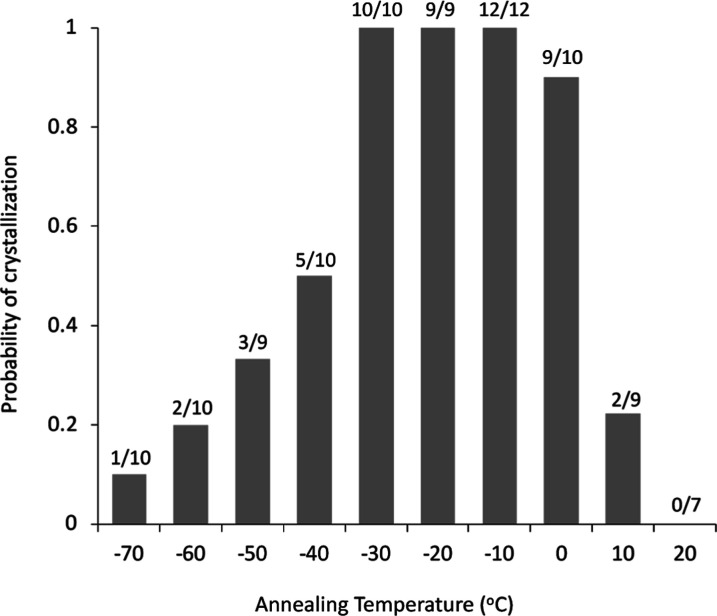
Probability of the cold crystallization of IBP glass after annealing at various temperatures for 1 h. The numbers on the bar graph show the frequency of crystallization. *a*/*b* represents the ratio of the trial and crystallization frequencies at *b* and *a* times, respectively.

**Figure 3:**
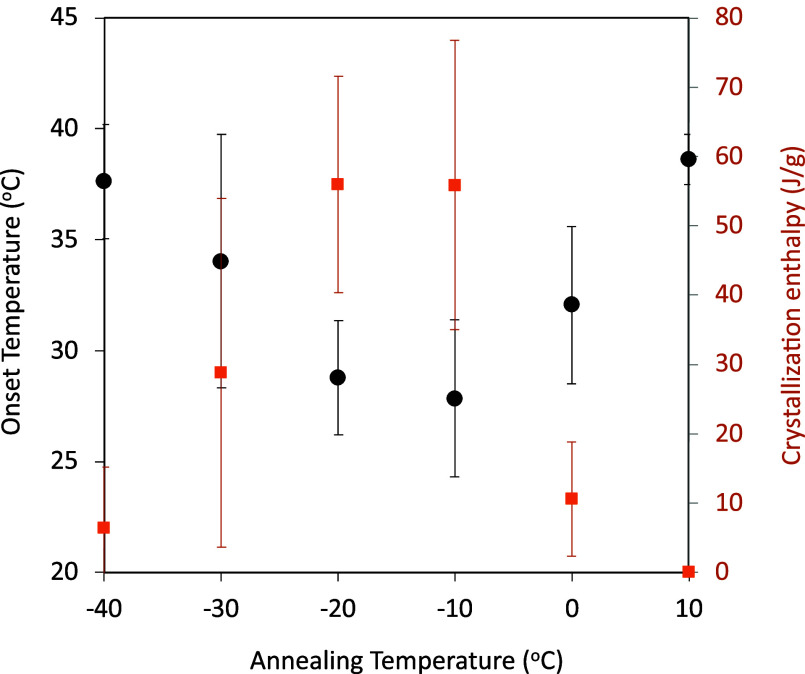
Onset temperature and crystallization enthalpy of IBP glass after annealing at various temperatures for 1 h. The number of experiments is reported in Figure 2. The data are presented as mean values with standard deviations (error bars).

Figure 2 shows the probability of cold crystallization after annealing at various temperatures for 1 h. Crystallization during the annealing was not observed for any annealing conditions as proved by compensatable relationship between cold crystallization and melting enthalpies.^21^ Cold crystallization during the DSC heating was consistently observed, when the annealing was performed at −30, −20, or −10 °C. In contrast, the probability of the cold crystallization significantly decreased with both an increase and a decrease in the annealing temperature. Figure 3 shows the onset temperature and crystallization enthalpy of the IBP glass after annealing at various temperatures for 1 h. Deviations of the crystallization enthalpy was significantly large for all annealing temperatures, indicating large variation in the number of nuclei formed during the annealing. The minimum and maximum values of the onset temperature and crystallization enthalpy, respectively, were obtained when the glass was annealed at −10 or −20 °C. Relationship between the cold crystallization enthalpy, Δ*H*_c_, and the melting enthalpy, Δ*H*_m_, can be described by the following equation.
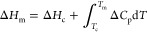
3where *T*_c_, *T*_m_, and Δ*C*_p_ are the cold crystallization temperature, the melting temperature, and difference in the specific heat capacity of glassy and the super-cooled liquid states. Δ*H*_c_ can be calculated as ca. 122 J/g by using Δ*H*_m_ = 135 J/g, *T*_c_ = 45 °C, *T*_m_ = 76 °C, and Δ*C*_p_ = 0.41 J/(g °C). Thus, averaged crystallinity achieved after the annealing at −10 or −20 °C for 1 h followed by the subsequent heating was below 50%.

**Figure 4:**
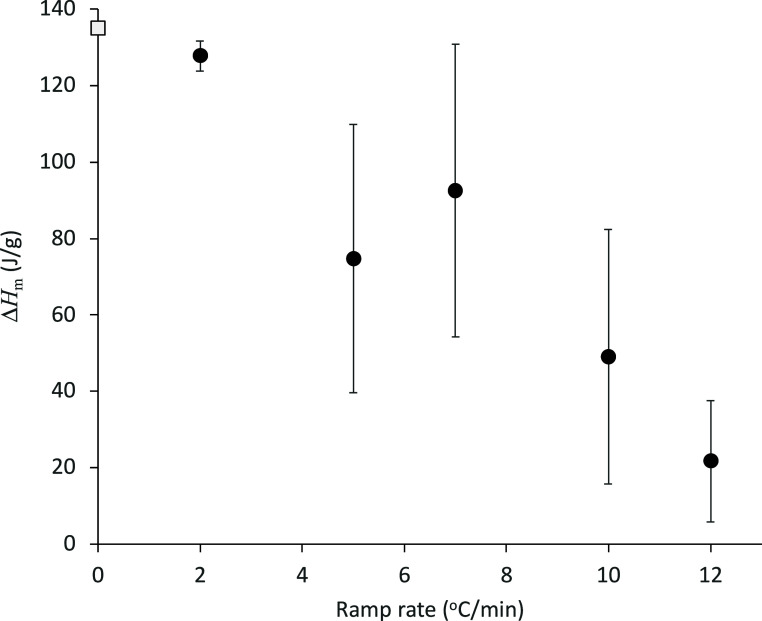
Melting enthalpy after the cold crystallization of IBP glass as a function of the ramp rate. The glasses were annealed at −20 °C for 1 h before the heating. The open square shows the melting enthalpy of the intact IBP crystal.

As crystal growth proceeded only during the heating process, the ramp rate significantly influenced the resultant crystallinity significantly. Figure 4 shows the melting enthalpy after cold crystallization as a function of the ramp rate, when the glass was annealed at −20 °C. The increase of the melting enthalpy was clearly observed with decreasing ramp rate to reach almost 100% crystallinity at a ramp rate of 2 °C/min.

**Figure 5:**
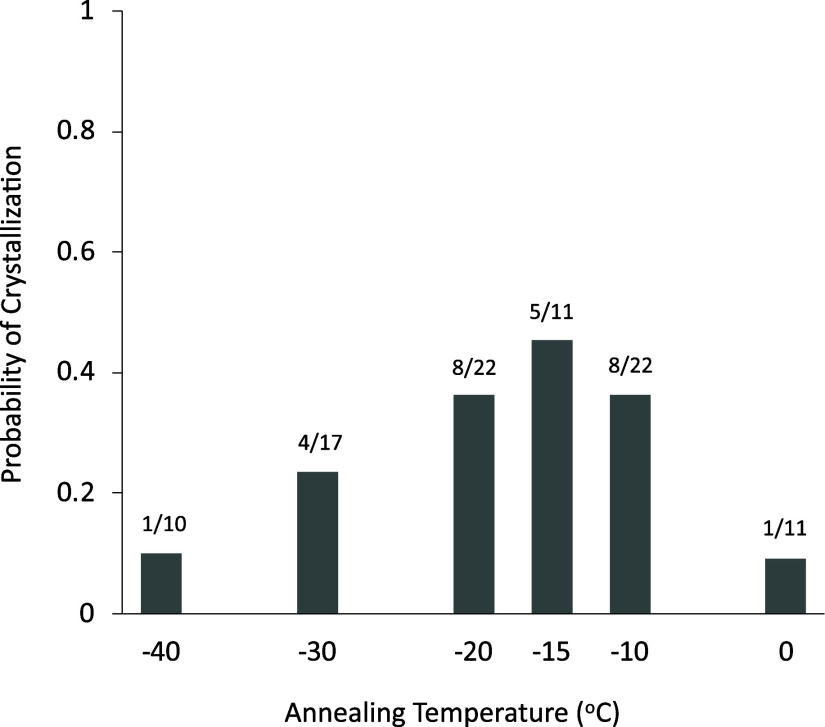
Probability of the cold crystallization of IBP glass after annealing at various temperatures for 20 min. The numbers on the bar graph show the frequency of crystallization. *a*/*b* represents the ratio of the trial and crystallization frequencies at *b* and *a* times, respectively.

For acquiring better knowledge of the nucleation temperature range that provides the highest probability, the annealing time was shortened to 20 min. Figure 5 shows the effect of the annealing temperature on the probability of cold crystallization, where the probability was found to decrease by decreasing the annealing time to 20 min. As annealing at −10 and −20 °C had the same probabilities, annealing was also conducted at −15 °C, which resulted in a higher probability of crystallization. Therefore, −15 °C was determined to be the optimum nucleation temperature for IBP glass. Thus, this entropic approach, that is, a comparison of the probability of cold crystallization, was proven to work well in determining the nucleation temperature range that provides highest probability of nucleation.

### 3.3 Crystallization Behavior of the IBP/Polymer Binary Mixture

**Figure 6:**
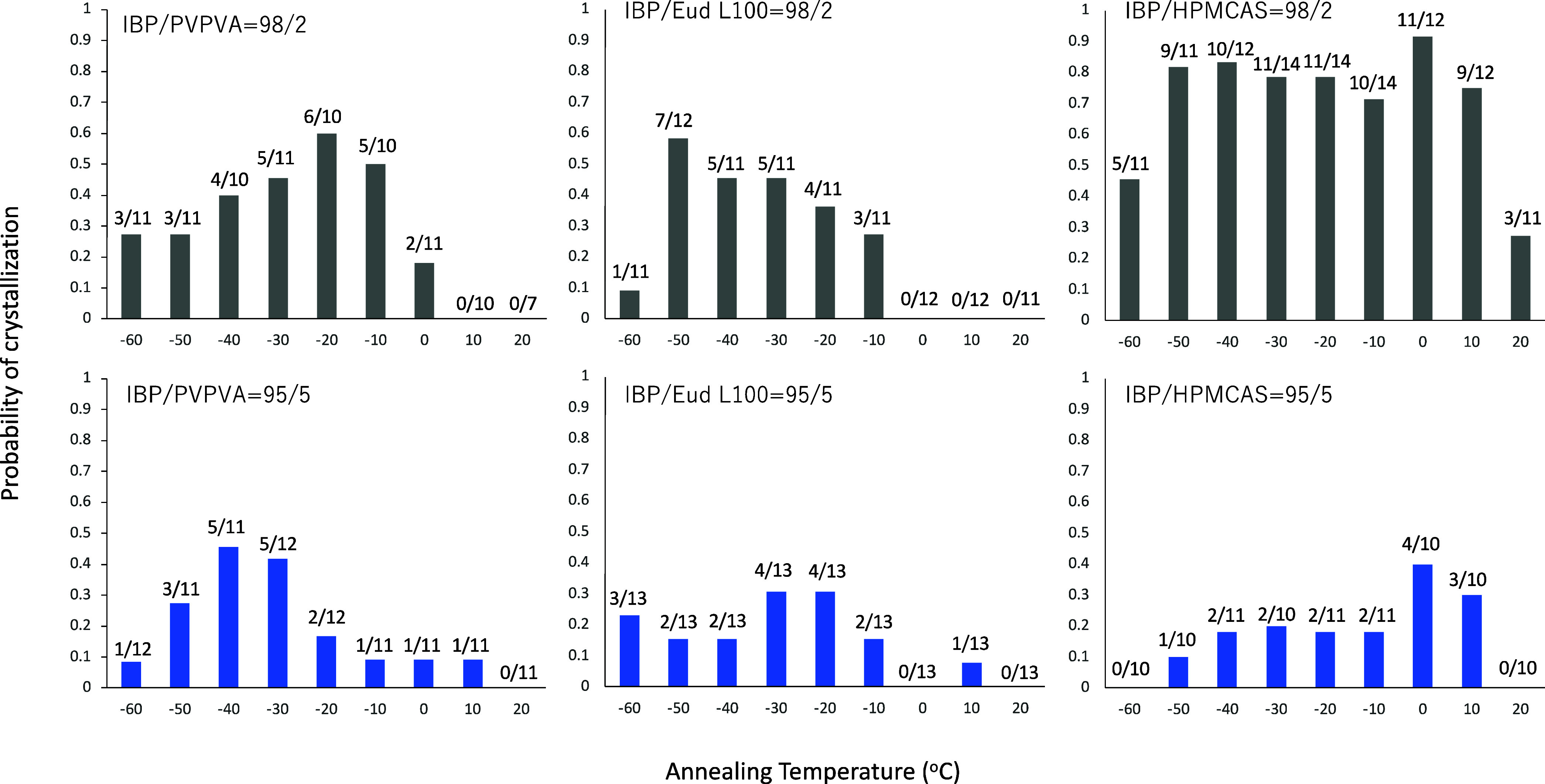
Probability of the cold crystallization of IBP glasses mixed with small amounts of polymer after annealing at various temperatures for 1 h. The numbers on the bar graph show the frequency of crystallization, which is presented in the same manner as those for Figure 2. The compositions are presented in the figures.

Pharmaceutical amorphous solid dispersions typically contain much higher amounts of polymeric additives than do drugs. Their stabilization mechanism includes an increase in the glass transition temperature (i.e., decrease in molecular mobility), interaction between the polymer and drug molecules, and the steric hindrance for crystallization by the polymer. The addition of a very small amount of polymer can significantly alter crystallization behavior.^22^ DSC second heating curves of the quenched mixtures of IBP and polymers are presented in the Supporting Information, where only one *T*_g_ was found for all samples. Thus, all mixtures were assumed to be mixed homogeneously after the quenching. Figure 6 shows the crystallization probabilities of IBP glasses quenched with 2% or 5% polymeric additives. Even the addition of 2% of PVPVA or Eud L100 was found to suppress the crystallization significantly. Moreover, the addition of Eud L100 or HPMCAS altered the range of optimum nucleation temperatures. In the presence of Eud L100, the optimum temperature was likely to be lowered to ca. −50 °C, whereas the nucleation temperature range was widened in the presence of HPMCAS. As probability of cold crystallization was 100% for pure IBP after the annealing at −30, −20, and −10 °C, the probability decreased a little in the presence of 2% HPMCAS. However, significant increase in the probability was observed for the annealing at −50, −40, and 10 °C. The addition of 5% polymer was found to suppress the crystallization significantly, regardless of the polymer type. The similar widening and depression of the nucleation temperature region was also reported for acetaminophen glass in the presence of polymers.^22^

**Figure 7:**
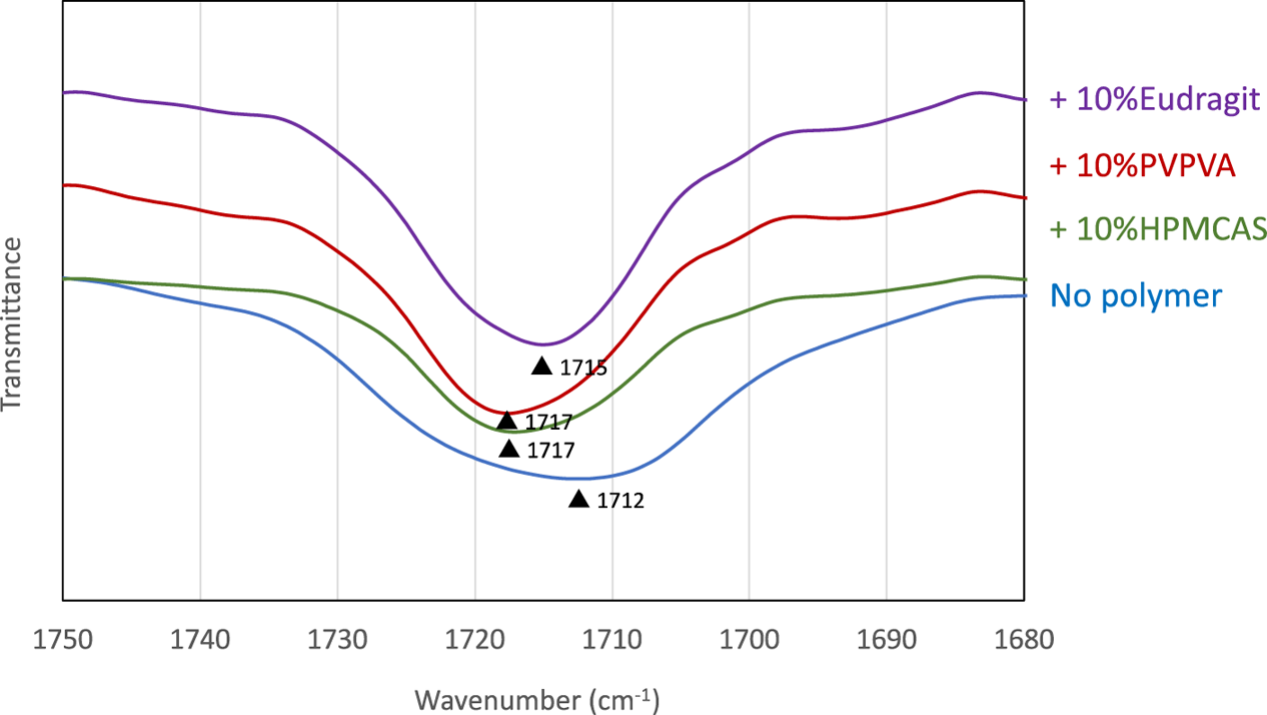
FT-IR spectra of the carbonyl region of the IBP glass quenched with 10% polymers. The peak wavenumbers are indicated by the triangles.

Figure 7 shows the change in the FT-IR spectra of IBP glass after the addition of 10% polymer. The higher polymer content relative to that for the crystallization study was employed for stressing the change in the FT-IR spectra caused by the addition of the polymers. The spectra in the presence of 5% polymer are presented in Supporting Information, which showed a smaller shift, but the trend was the same. It is generally believed that the polymers that interact strongly with the drug possess strong inhibitory effect for crystallization.^23,24^ IBP has both hydrogen-bond donors and acceptors. All polymers induced a shift in the carbonyl region band to a higher wavelength, indicating the formation of hydrogen bonds between IBP and the polymer. The large shift was observed for HPMCAS and PVPVA. Thus, these polymers are expected to inhibit crystallization of IBP effectively; however, this agrees with the observation only partially.

**Figure 8:**
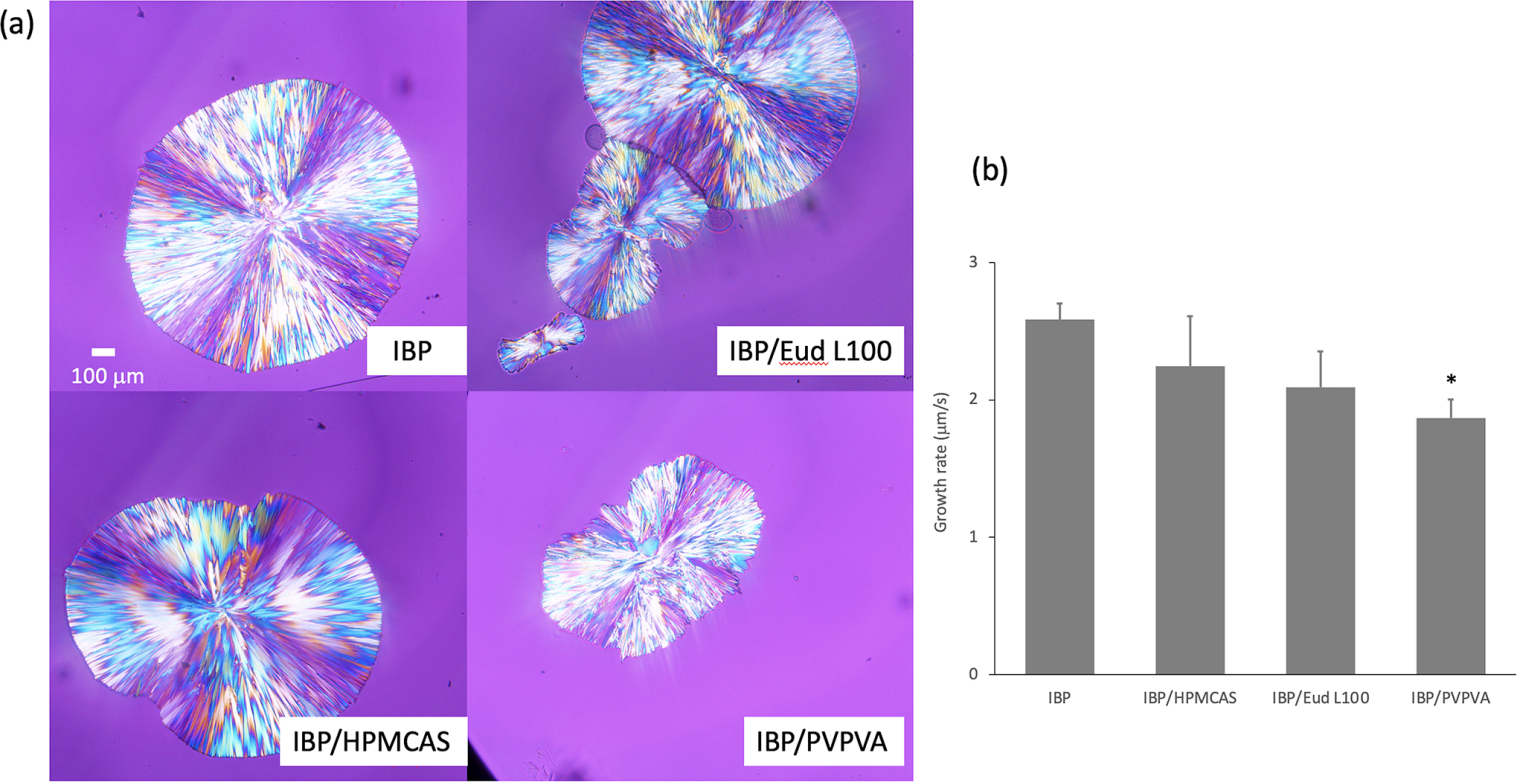
(a) Representative PLM images of the crystallization process of IBP glass at 40 °C. IBP/polymer = 98/2. The scale is the same for all images. (b) Crystal growth rates of IBP glass and its mixtures with polymers determined from the PLM images. The growth rates were determined for the crystals which do not have other crystals nearby, as it was likely to suppress the growth rate. * Statistically meaningful difference relative to IBP (*P* < 0.05).

The crystal growth process was investigated under PLM to clarify the inhibition mechanism of crystallization of IBP by polymers. Figure 8a shows representative images of the IBP crystals during the growth. Crystals grew in a spherical shape regardless of the absence or presence polymers. However, the shape was distorted in the presence of polymers presumably because of decrease in the interfacial tension. Moreover, the interface was rough in the presence of PVPVA, which also indicated lower interfacial tension. Determination of nucleation rate from the microscopic observation was difficult because only a small number of crystals appeared. However, although only one crystal was typically observed in the absence of polymers, multiple number of crystals were occasionally found in the presence of the polymer, suggesting the influence of the polymer on the nucleation rate. Especially, Eud L100 was likely to enhance the nucleation, as many crystals were frequently found to grow simultaneously in the observation. The comparison of growth rates (Figure 8b) revealed that the addition of 2% polymers was likely to suppress the growth rate; however, no statistically meaningless differences were found except for the addition of PVPVA.

**Figure 9:**
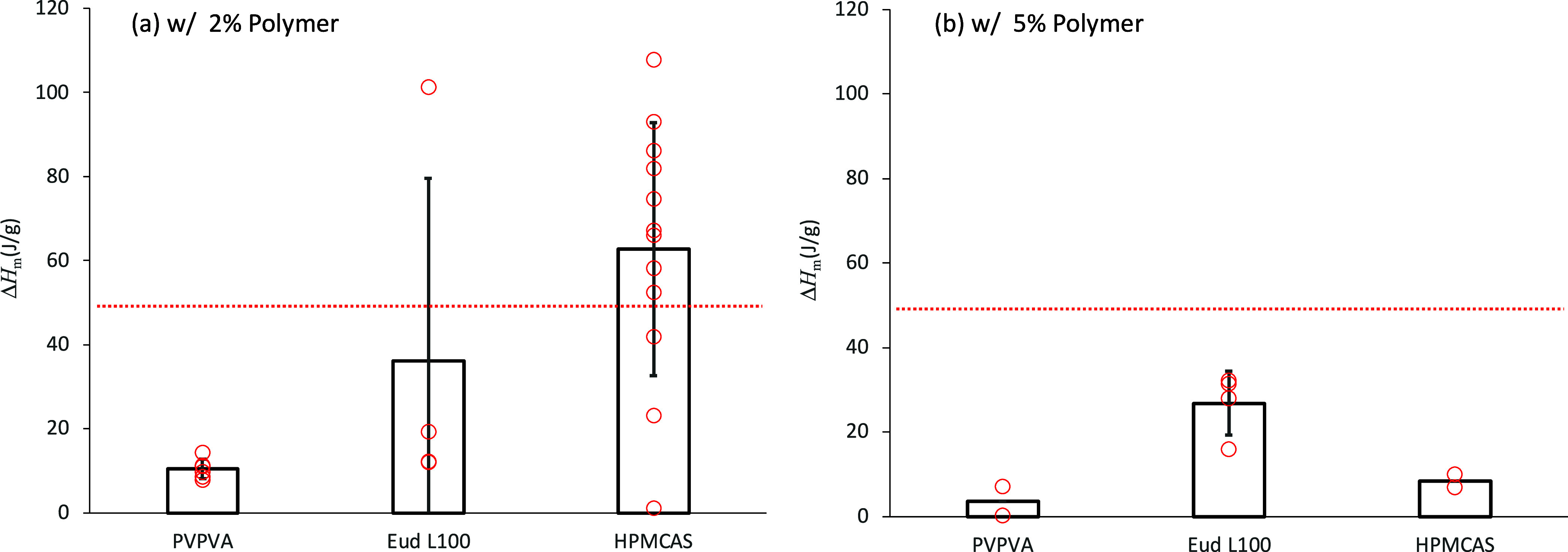
Effect of the coexisting polymer on the melting enthalpy of IBP crystallized after annealing at −20 °C for 1 h. IBP was mixed with (a) 2% or (b) 5% of polymer. Both individual data (red open circles) and the averaged values (bar graph, with standard deviations as error bars) are presented. The red line is the mean melting enthalpy of the IBP glass crystallized and melted without the polymer under the same condition.

The melting enthalpy of the cold crystallized IBP in the presence of polymers is summarized in Figure 9. In the presence of the 2% PVPVA or Eud L100, the melting enthalpy was significantly reduced except for one case in the presence of Eud L100. This was not observed in the presence of 2% HPMCAS. In the presence of 5% of polymers, the melting enthalpy was reduced regardless of the polymer type. The reduction in the melting enthalpy, which is associated with reduction in the cold crystallization enthalpy as well, may be explained by suppression of the crystal growth and/or decreased number of the nuclei. The decreased melting enthalpy in the presence of PVPVA may be explained by a suppressed nucleation rate, as revealed by the PLM observation (Figure 8b). However, it cannot be the only reason for other polymers, as the widening and depression of the nucleation temperature region, in the presence of HPMCAS and Eud L100, respectively (Figure 6), are difficult to explain by the inhibitory effect for the crystal growth. Therefore, the added polymers are the most likely to influence the nucleation process.

### 3.4 Effect of Polymer on Glass Dynamics

**Figure 10:**
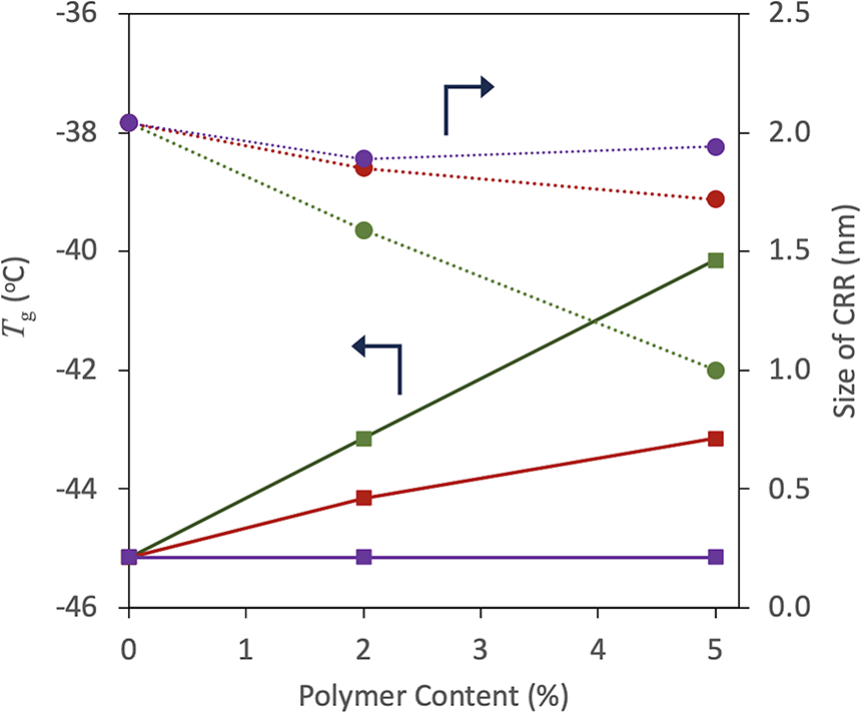
Effect of addition of polymers on *T*_g_ and size of CRR of the IBP glass at *T*_g_. Circles and squares stand for size of CRR and *T*_g_, respectively. Polymer types: Eud L100 (purple), PVPVA (red), and HPMCAS (green). The error bars based on the standard deviation for *T*_g_ were smaller than the symbols.

Figure 10 shows the effect of the added polymer on *T*_g_ and size of CRR of IBP glass at *T*_g_. The DSC curves at *T*_g_ are provided in the Supporting Information. The midpoint *T*_g_s of PVPVA, Eud L100, and HPMCAS were 108, 195, and 122 °C, respectively.^25,26^ Thus, the most effective increase in *T*_g_ was expected for the addition of Eud L100, which was followed by HPMCAS and PVPVA, if the polymer and the drug exhibit ideal mixing. However, *T*_g_ did not increase upon the addition of Eud L100, whereas the most effective increase was observed for the inclusion of HPMCAS, when the polymer amount was below 5%. This difference was originated from impact on the width of the glass transition region as presented in the Supporting Information, suggesting that influence on the molecular cooperativity significantly depended on the polymer type added. Therefore, the impact on the size of CRR showed a similar trend; that is, the most effective change was observed for the addition of HPMCAS, followed by PVPVA and Eud L100. The size of CRR in the presence of 5% HPMCAS, 1.0 nm, was nearly half of that for pure IBP. It is frequently observed that the addition of a small amount of Eudragit to pharmaceutical glasses rarely influenced *T*_g_.^22^ As Eudragit has an amphiphilic property, it may cause microphase separation when the added amount is small without affecting the dynamics of the IBP molecules.

The relationship between the size of the CRR and the nuclei is not understood well. However, it is reasonable to expect nucleation to occur effectively if the CRR size is similar to that of the nuclei. Under this assumption, the enhancement of crystallization in the presence of 2% HPMCAS may be understood by the coincidence of the CRR size with that of the nuclei. One possible explanation for the suppression of crystallization in the presence of 5% HPMCAS is a further decrease in the CRR size. Other possibilities include an increase in viscosity in the presence of polymers and their steric hindrance in inhibiting association of IBP molecules.

As the temperature dependence of the molecular cooperativity is related to the activation energy of the molecular dynamics,^27,28^ the size of CRR is expected to correlate with fragility.^29,30^ The fragility of each mixture was determined from the heating rate dependence of *T*_g_ (Supporting Information). The fragility value of the IBP glass is 75.^20^ It changed to 72, 64, and 62, in the presence of 10% Eud L100, PVPVA, and HPMCAS, respectively. This is analogous to the finding that the addition of PVPVA or HPMCAS decreased the size of CRR, whereas Eud L100 did not. The impact on the FT-IR spectra of the carbonyl region also exhibited a similar trend (Figure 7), indicating the possible impact of hydrogen bonding on the CRR size. Thus, the influence of polymer addition on the crystallization behavior was not explained by the molecular interaction between IBP and the polymer; however, it was likely to affect indirectly through the influence on the CRR size of IBP glass.

### 3.5 Relevance to Classical Nucleation Theory

**Figure 11:**
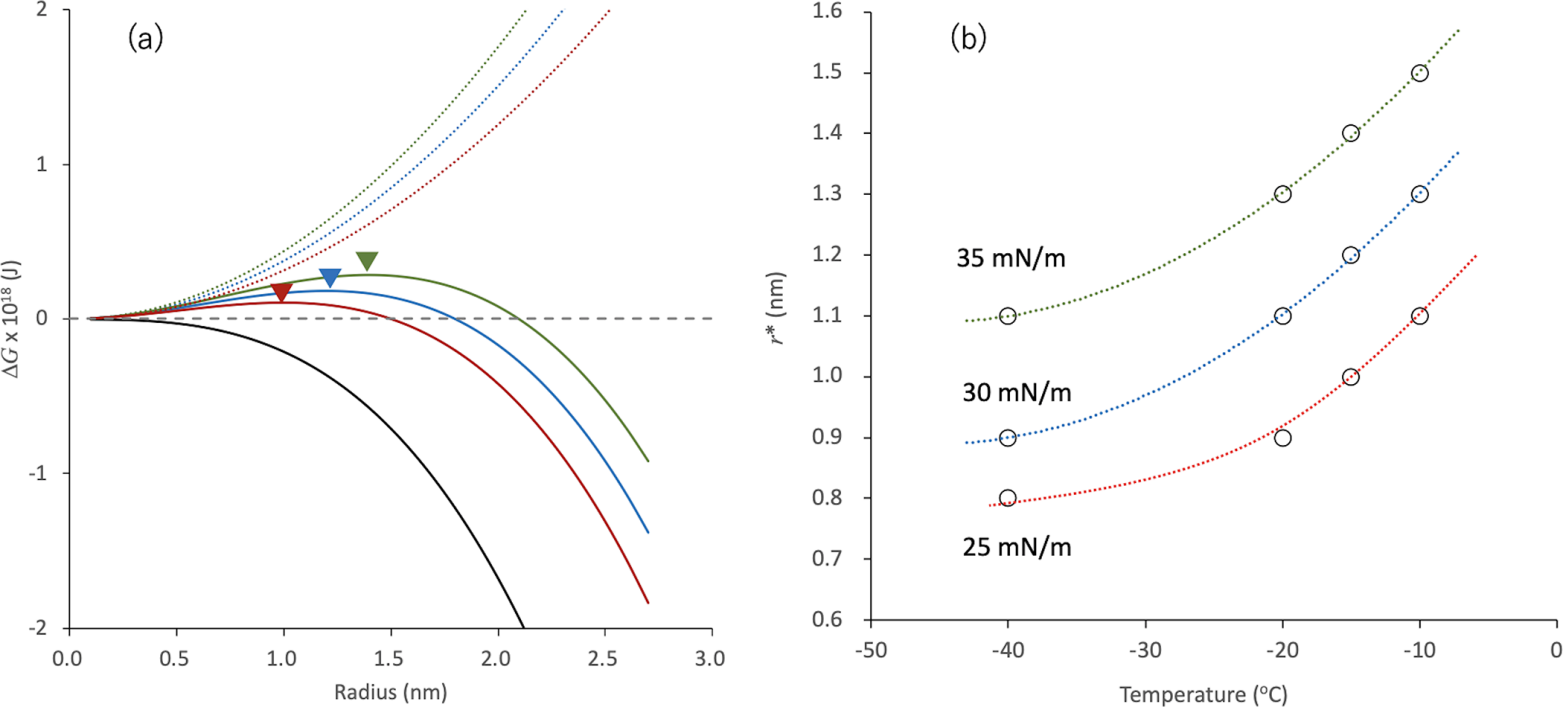
(a) Model calculations of Δ*G*_i_, (dotted, colored), Δ*G*_t_ (black) for IBP glass and their summation (solid, colored) as a function of radius of nuclei at −15 °C. The assumed interfacial tension values are 25 (red), 30 (blue), and 35 (green) mN/m. The triangles show the critical radius of nuclei (r*), where Δ*G*_i_ + Δ*G*_t_ exhibits the maximum. (b) Predicted r* values for IBP glass as a function of temperature. The interfacial tension values used for calculation is presented in the figure.

According to classical nucleation theory, the critical radius of nuclei, r*, is determined by the balance of the interfacial energy, Δ*G*_i_, and the thermodynamic transformation energy, Δ*G*_t_. Each contribution is described as follows.
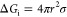
4
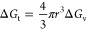
5Here, *r* and σ are the radius of the nuclei and interfacial energy, respectively. Δ*G*_v_ is the Gibbs energy difference between the crystal and the liquid, which can be calculated using the melting temperature (76 °C) and enthalpy (26.5 kJ/mol) of IBP.^7^ Interfacial tension between the nuclei and glass is difficult to evaluate. However, that between ice and water (ca. 30 mN/m)^31^ may work for temporal calculations. Figure 11a shows Δ*G*_i_, Δ*G*_t_ for IBP glass, and their summation as a function of the radius of nuclei at −15 °C, which revealed that r* for IBP glass was ca. 1.2 nm. As this value is close to the size range for the CRR of IBP/HPMCAS glass, this model calculation supports the idea that crystallization is more likely to proceed when the size of the CRR is close to r*. As the interfacial tension value used for the calculation was uncertain, other values of 25 and 35 mN/m were also used for the calculation to determine r* (Figure 11b), where the dependence of r* on the temperature was also evaluated. The deviation in r* ranged from 1.0 to 1.4 nm at −15 °C, which is consistent with the size range of the CRR of the IBP glass. The addition of the polymer may reduce the interfacial tension. If this happens, r* becomes smaller than that for pure IBP. In fact, the shift of the nucleation temperature to a lower temperature in the presence of the polymer was found for the addition of PVPVA and Eud L100 (Figure 6).

The maximum nucleation rate is a result of competition between the kinetic and thermodynamic barriers, which decrease and increase with increasing temperature, respectively. However, this assumption is not sufficient to explain the nucleation temperature found above *T*_g_ because the thermodynamic driving force of nucleation continues to increase monotonically with decreasing temperature.^32^ One idea to solve this problem was to assume “structural units” to explain a slower diffusion rate than expected,^32^ which may be analogous to CRR. Although further verification based on theoretical and experimental evidence is required, attention to CRR may explain various unexplainable phenomena related to crystallization.

In summary, polymer molecules affect the crystallization process in various manners including decrease in glass/nucleus interfacial tension to assist nucleation and increase in viscosity to suppress crystal growth. Influence on the CRR size may also be important. Although none of them seemed to be negligible, only the change in the CRR size could explain the opposite effect on the crystallization probability depending on the added amount of polymer, as seen for the 2% and 5% HPMC addition (Figure 6).

## 4 Conclusions

The nucleation temperatures of pharmaceutical glasses must be understood to ensure their physical stability. In this study, an entropic approach was used to identify the nucleation temperature of the IBP glass. The probability of cold crystallization was determined after annealing the IBP glass at various temperatures to find the optimum nucleation temperature for Form I at −15 °C. The addition of a small amount of polymer suppressed the crystallization of IBP in most cases; however, the acceleration of crystallization was found when 2% HPMCAS was added. The effect of polymers on the crystallization behavior was explained by their direct interaction with IBP, increase in viscosity to suppress crystal growth, and alteration of the CRR size. The opposite effect on the crystallization probability depending on the added amount of polymer could be explained only by the impact on the CRR size, as seen for the 2% and 5% HPMCAS addition. This viewpoint may provide a better understanding and practical guidance for controlling crystallization behavior of glass materials.

## Supporting Information Available

Examples of DSC heating curves of IBP glass annealed at −50 or −10 °C for 1 h; DSC heating curves of IBP/polymer mixtures and polymers; glass transition of IBP and its mixtures with polymers investigated by modulated-temperature DSC; DSC curves for determining fragility of IBP glass and its mixtures with polymers; and FT-IR measurements in the presence of 5% polymer (PDF)
