# Supplementary material for: Crystal Nucleation in Ibuprofen Glass: Possible Relevance between the Characteristic Length of the Cooperatively Rearranging Region and the Size of Crystal Nuclei
Source: J Phys Chem B. 2025 Feb 6;129(7):2096–104. doi: 10.1021/acs.jpcb.4c07005 (PMC11848918; doi:10.1021/acs.jpcb.4c07005)
Supplement: Supplementary file 1 — jp4c07005_si_001.pdf [file jp4c07005_si_001.pdf]

Supporting information for

# Crystal Nucleation in Ibuprofen Glass: Possible Relevance between Characteristic Length of Cooperatively Rearranging Region and Size of Crystal Nuclei

Kohsaku Kawakami[1,2,*] and Kaoru Ohyama

[1] Research Center for Macromolecules and Biomaterials, National Institute for Materials Science, 1-1 Namiki, Tsukuba, Ibaraki 305-0044, Japan

[2] Graduate School of Pure and Applied Sciences, University of Tsukuba, 1-1-1 Tennodai, Tsukuba, Ibaraki 305-8577, Japan

## 1. DSC heating curves of IBP glass annealed at -50 or -10 °C for 1h.

Below presented are examples of the DSC heating curves of IBP glass annealed at -50 or -10 °C for 1h. The cold crystallization was always observed after annealing at -10 °C; however, it was not the case after the annealing at -50 °C.

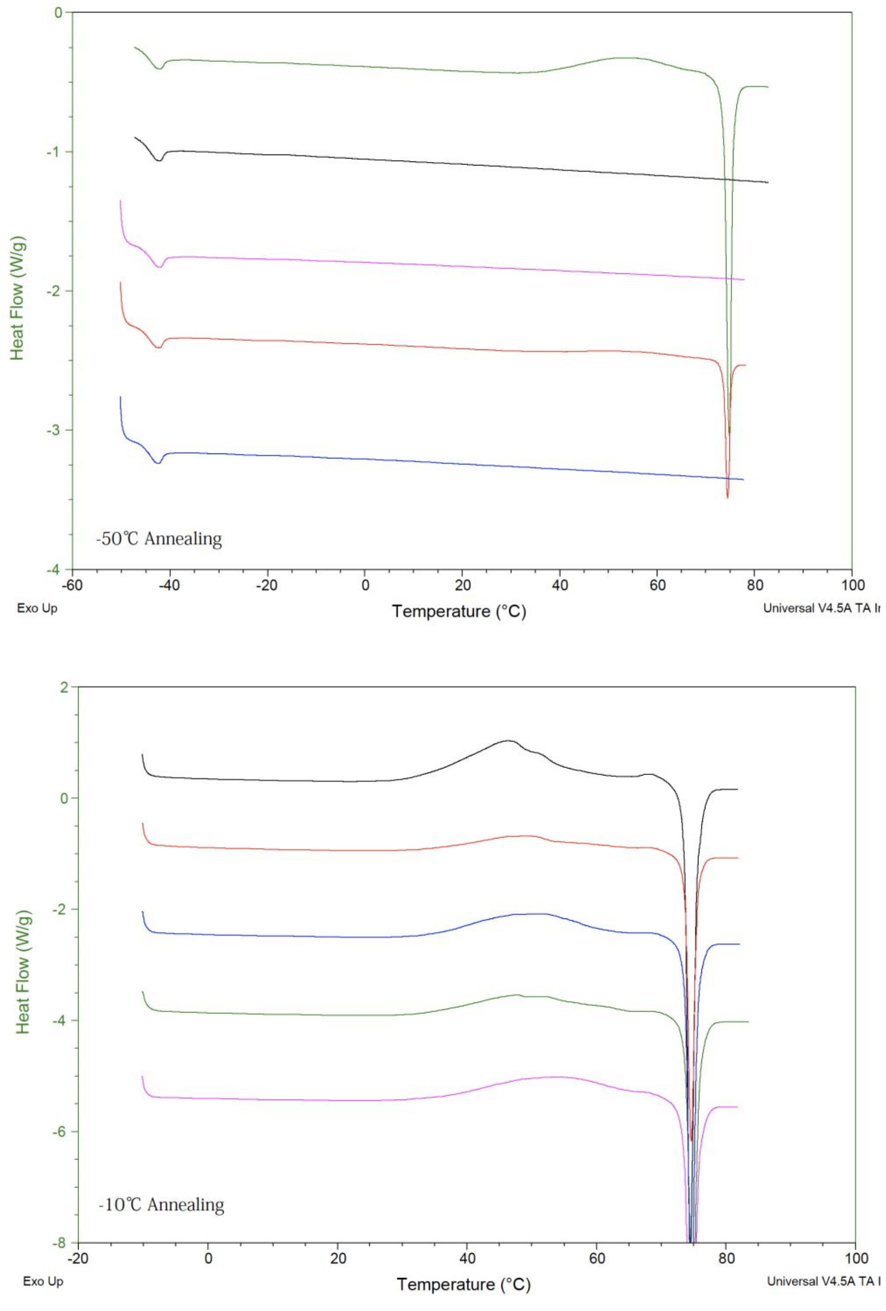


Figure S1 Examples of the DSC heating curves of IBP glass annealed at -50 °C (upper) or -10 °C (lower) for 1h.

## 2. DSC Heating Curves of IBP/Polymer Mixtures

The mixtures of IBP and polymers (PVPVA, HPMCAS, and Eudragit) were prepared by grinding followed by melt-quench in DSC. Below presented are the second DSC heating curves of the quenched samples. Also presented are DSC curves of pure PVPVA and HPMCAS. $T_g$ of Eudragit L100 is reported to be 195 °C (Parikh et al., J. Excipients and Food Chem. 2014, 5, 56-64). As this is above its decomposition temperature, we did not make attempt to acquire the data. As shown below, only one $T_g$ was found for all the mixtures. Thus, all mixtures are supposed to be homogeneously mixed.

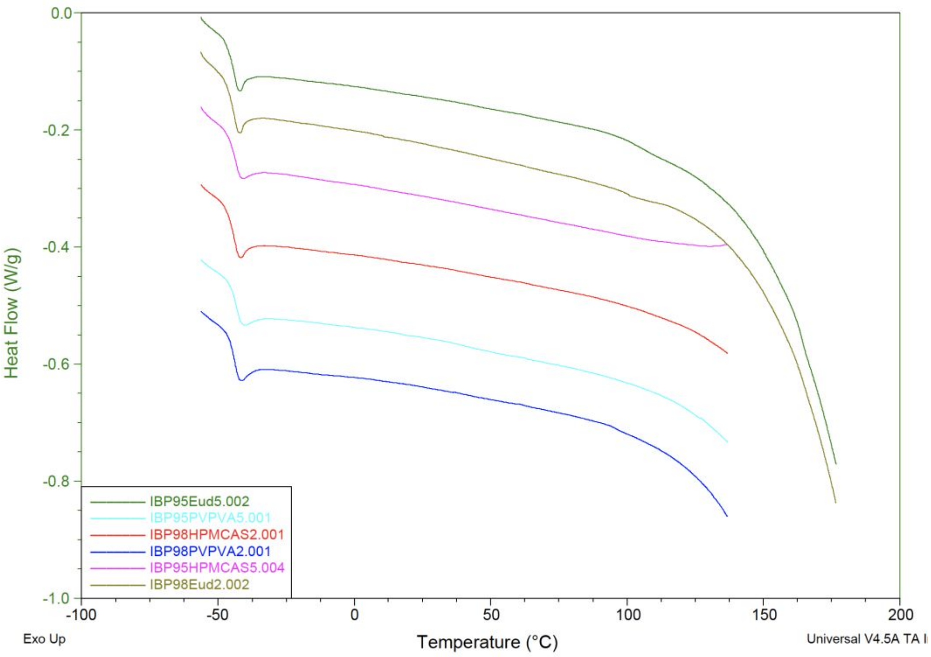

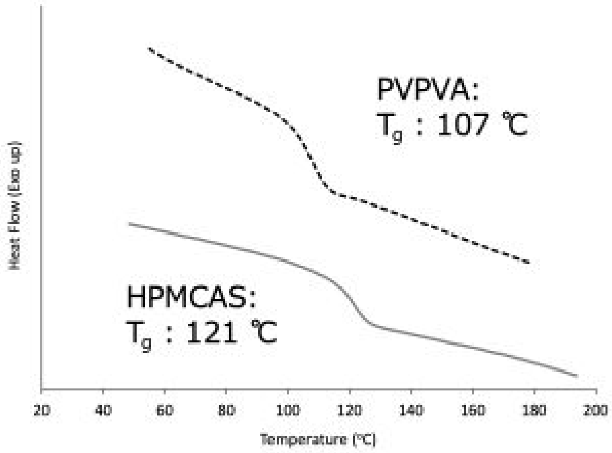


Figure S2 (upper) The second DSC heating curves of the quenched samples. Compositions are presented in the figure. (lower) DSC curves of pure PVPVA and HPMCAS

## 3. The glass transition of IBP and its mixtures with polymers investigated by modulated-temperature DSC

Below presented is the reversing heat capacity curves in the glass transition region of IBP glass and its mixtures with the polymers. The absolute heat capacity values and width of the glass transition region were determined from these curves to calculate size of CRR using eq. (1) in the main text.

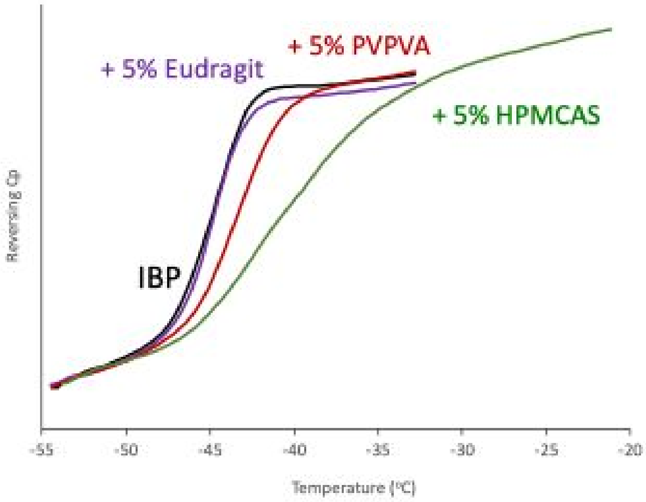

Figure S3 Reversing heat capacity curves in the glass transition region of IBP glass and its mixtures with the polymers.

Table S1 Parameters for calculating size of CRR

| | $T_g$ (K) | $\Delta d_{Tg}$ (°C) | $C_{pg}$ (J/g°C) | $C_{pl}$ (J/g°C) | $L$ (nm) |
|---|---|---|---|---|---|
| IBP | 228 | 1.98 | 1.10 | 1.54 | 2.04 |
| 2%HPMCAS | 230 | 2.47 | 1.17 | 1.51 | 1.59 |
| 5%HPMCAS | 233 | 5.08 | 1.21 | 1.58 | 1.00 |
| 2%PVPVA | 229 | 2.03 | 1.16 | 1.52 | 1.85 |
| 5%PVPVA | 230 | 2.35 | 1.11 | 1.47 | 1.72 |
| 2%Eud L100 | 228 | 1.97 | 1.15 | 1.51 | 1.89 |
| 5% Eud L100 | 228 | 1.85 | 1.17 | 1.52 | 1.94 |

## 4. Fragility of IBP glass and its mixtures with polymers

Fragility of IBP glass and its mixtures with polymers was determined from heating rate dependence of $T_g$. The glass transition behaviors during the DSC heating are presented below for each mixture. Using these $T_g$ values (n=3), fragility was determined using the procedure described in the main text.

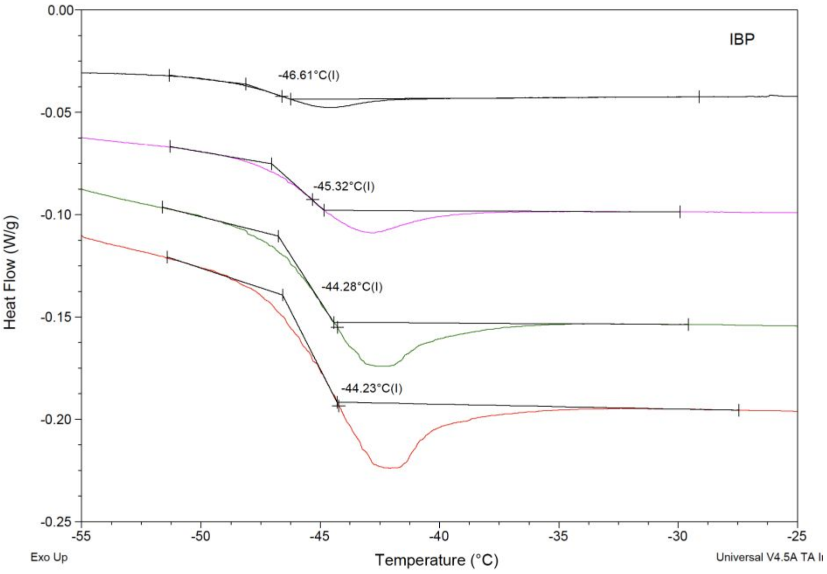
IBP
-46.61°C(I)
-45.32°C(I)
-44.28°C(I)
-44.23°C(I)
Heat Flow (W/g)
Temperature (°C)
Exo Up
Universal V4.5A TA I

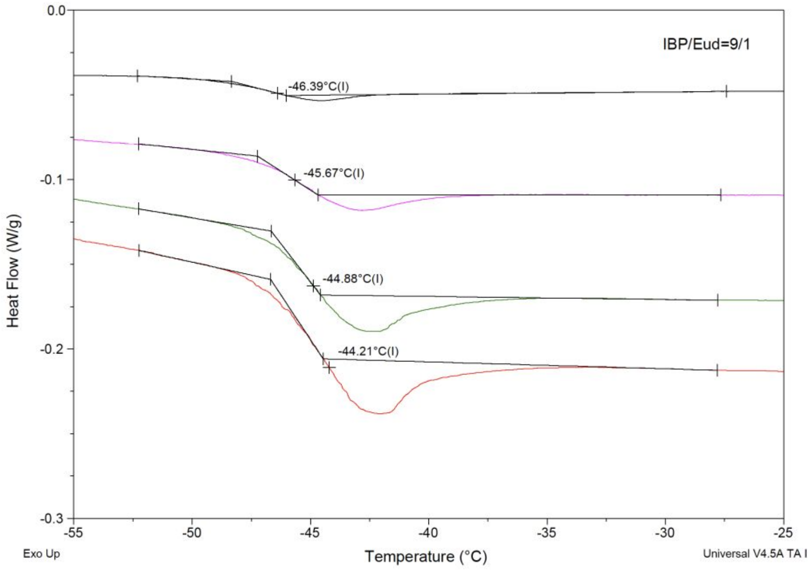
IBP/Eud=9/1
-46.39°C(I)
-45.67°C(I)
-44.88°C(I)
-44.21°C(I)
Heat Flow (W/g)
Temperature (°C)
Exo Up
Universal V4.5A TA I

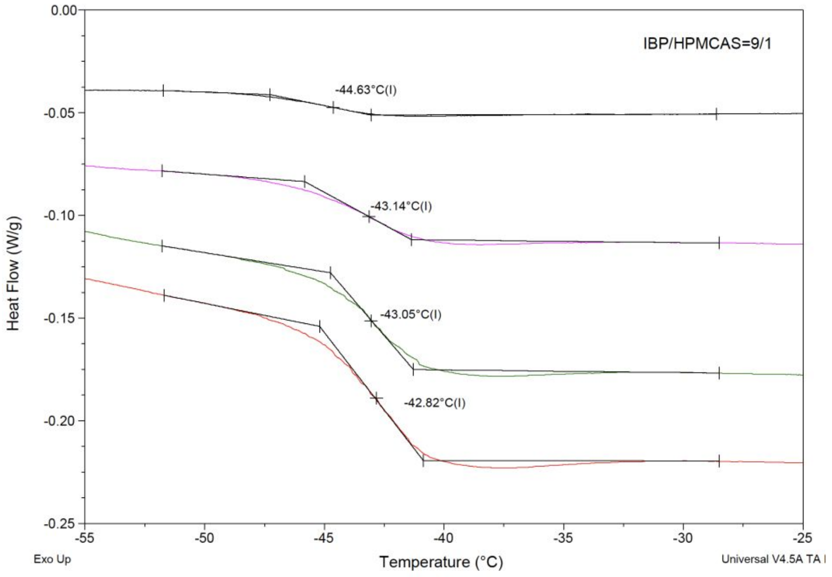


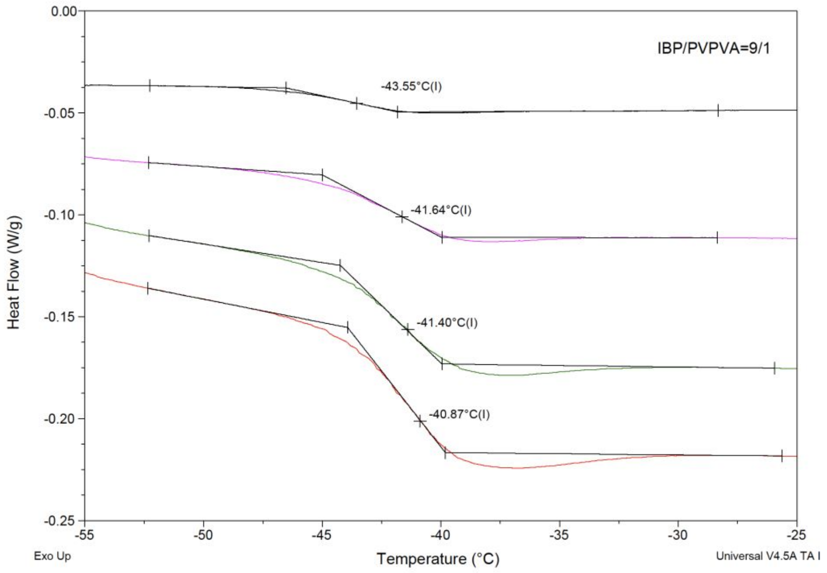


Figure S4 The glass transition behaviors during the DSC heating. Compositions are presented in the figure. The heating rates are 2, 5, 8, and 10 °C/min (top to bottom).

## 5. FT-IR measurements in the presence of 5% polymer.

In the main text, the FT-IR spectra of IBP in the presence of 10% polymer is presented for stressing the change cause by the polymers. Below presented is the FT-IR spectra in the presence of 5% polymer, which was subjected to the crystallization study. The shift

was smaller compared to the addition of the 10% polymer, but the trend remained the same.

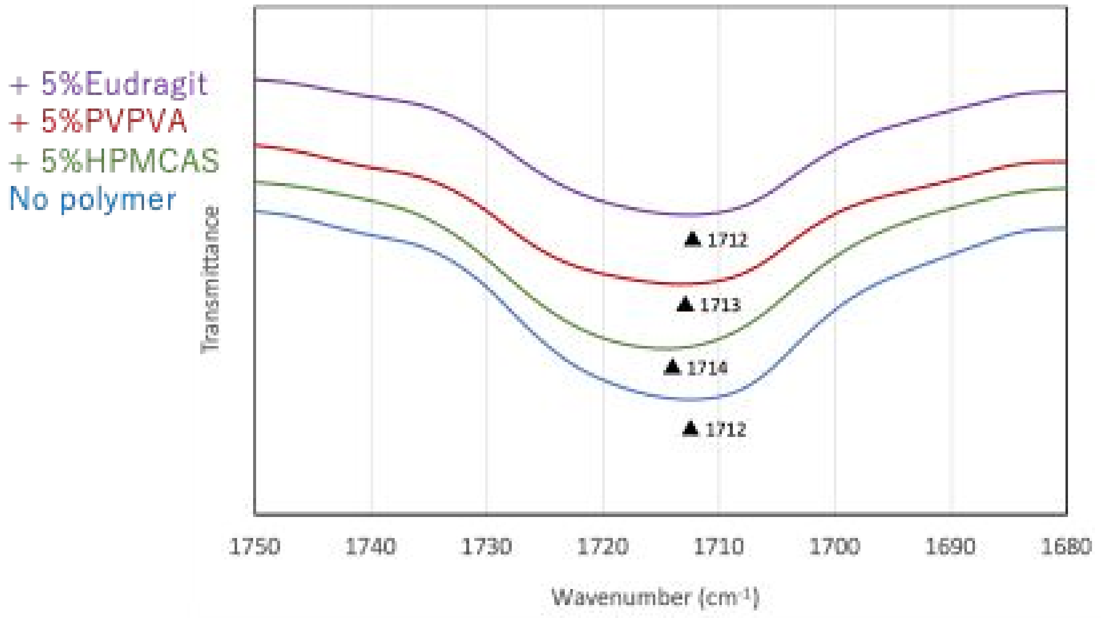


Figure S5 FT-IR spectra of IBP glass and IBP/polymer = 95/5 glasses.
